# The pivotal role of *aristaless* in development and evolution of diverse antennal morphologies in moths and butterflies

**DOI:** 10.1186/s12862-018-1124-2

**Published:** 2018-01-25

**Authors:** Toshiya Ando, Haruhiko Fujiwara, Tetsuya Kojima

**Affiliations:** 1 Department of Integrated Biosciences, Graduate School of Frontier Sciences, The University of Tokyo, Kashiwa, Chiba, 277-8562 Japan; 2 Present address: Division of Evolutionary Developmental Biology, National Institute for Basic Biology, Okazaki, Aichi 444-8585 Japan

**Keywords:** *aristaless*, antenna, morphological evolution, Lepidoptera

## Abstract

**Background:**

Antennae are multi-segmented appendages and main odor-sensing organs in insects. In Lepidoptera (moths and butterflies), antennal morphologies have diversified according to their ecological requirements. While diurnal butterflies have simple, rod-shaped antennae, nocturnal moths have antennae with protrusions or lateral branches on each antennal segment for high-sensitive pheromone detection. A previous study on the *Bombyx mori* (silk moth) antenna, forming two lateral branches per segment, during metamorphosis has revealed the dramatic change in expression of antennal patterning genes to segmentally reiterated, branch-associated pattern and abundant proliferation of cells contributing almost all the dorsal half of the lateral branch. Thus, localized cell proliferation possibly controlled by the branch-associated expression of antennal patterning genes is implicated in lateral branch formation. Yet, actual gene function in lateral branch formation in *Bombyx mori* and evolutionary mechanism of various antennal morphologies in Lepidoptera remain elusive.

**Results:**

We investigated the function of several genes and signaling specifically in lateral branch formation in *Bombyx mori* by the electroporation-mediated incorporation of siRNAs or morpholino oligomers. Knock down of *aristaless*, a homeobox gene expressed specifically in the region of abundant cell proliferation within each antennal segment, during metamorphosis resulted in missing or substantial shortening of lateral branches, indicating its importance for lateral branch formation. *aristaless* expression during metamorphosis was lost by knock down of *Distal-less* and WNT signaling but derepressed by knock down of Notch signaling, suggesting the strict determination of the *aristaless* expression domain within each antennal segment by the combinatorial action of them. In addition, analyses of pupal *aristaless* expression in antennae with various morphologies of several lepidopteran species revealed that the *aristaless* expression pattern has a striking correlation with antennal shapes, whereas the segmentally reiterated expression pattern was observed irrespective of antennal morphologies.

**Conclusions:**

Our results presented here indicate the significance of *aristaless* function in lateral branch formation in *B. mori* and imply that the diversification in the *aristaless* expression pattern within each antennal segment during metamorphosis is one of the significant determinants of antennal morphologies. According to these findings, we propose a mechanism underlying development and evolution of lepidopteran antennae with various morphologies.

**Electronic supplementary material:**

The online version of this article (10.1186/s12862-018-1124-2) contains supplementary material, which is available to authorized users.

## Background

**Fig. 1:**
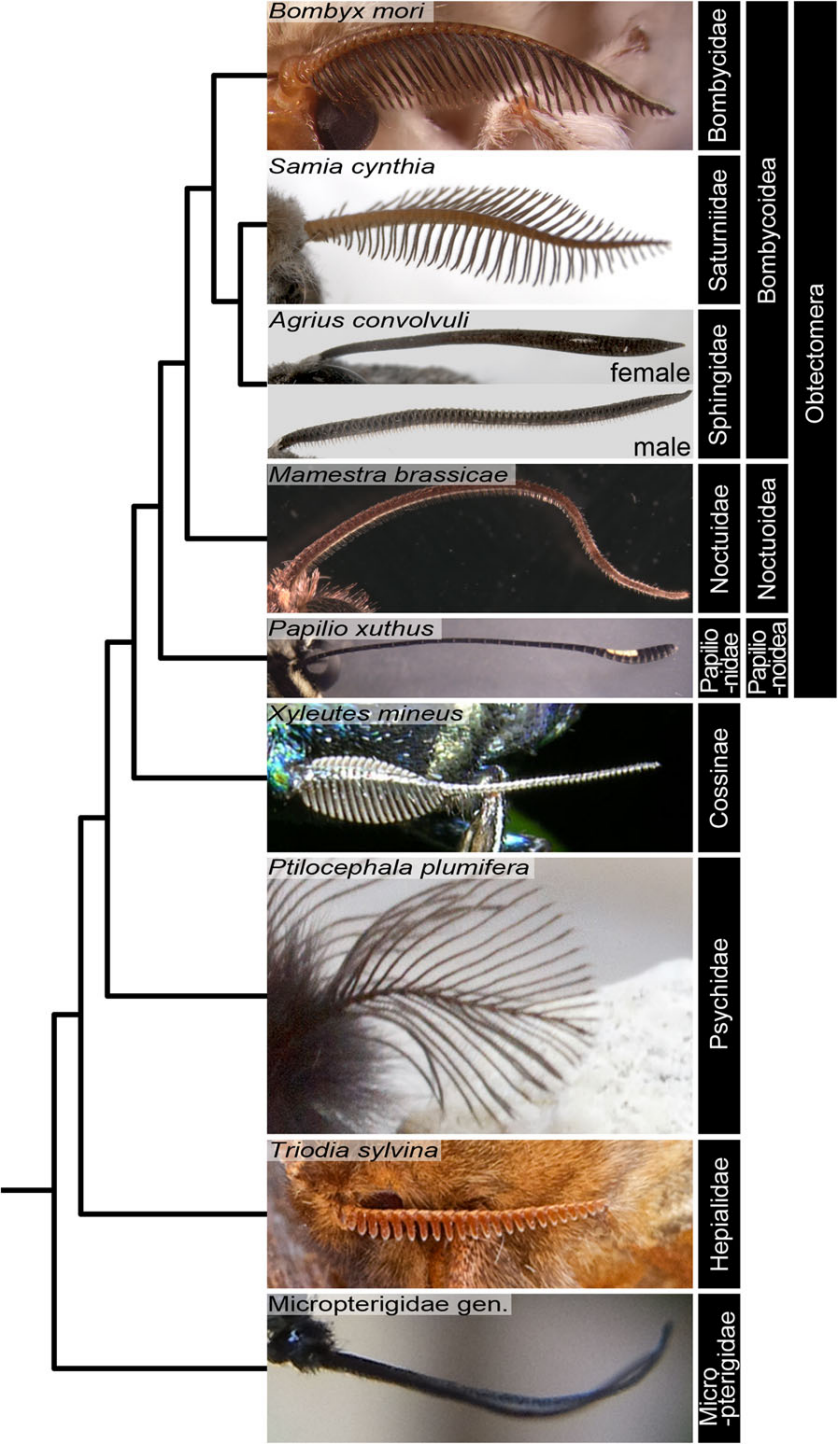
Various antennal morphologies in Lepidoptera. Examples of antennae with various morphologies in various species of Lepidoptera are presented. Note that antennae associated with branches or protrusions occur in several independent lineages within Lepidoptera. The unscaled phylogenetic tree shown in the left side of photographs is based on the maximum likelihood tree by Regier et al. [49]. The tree form concerning the shown species is consistent with that of the recent more probable tree by Kawahara et al. except for the Psychidae not included in the analysis [7]. Species name are presented at upper-left corner and those of family, superfamily and subclade are indicated in the right side of photographs. Magnifications of photographs are arbitrary

**Fig. 2:**
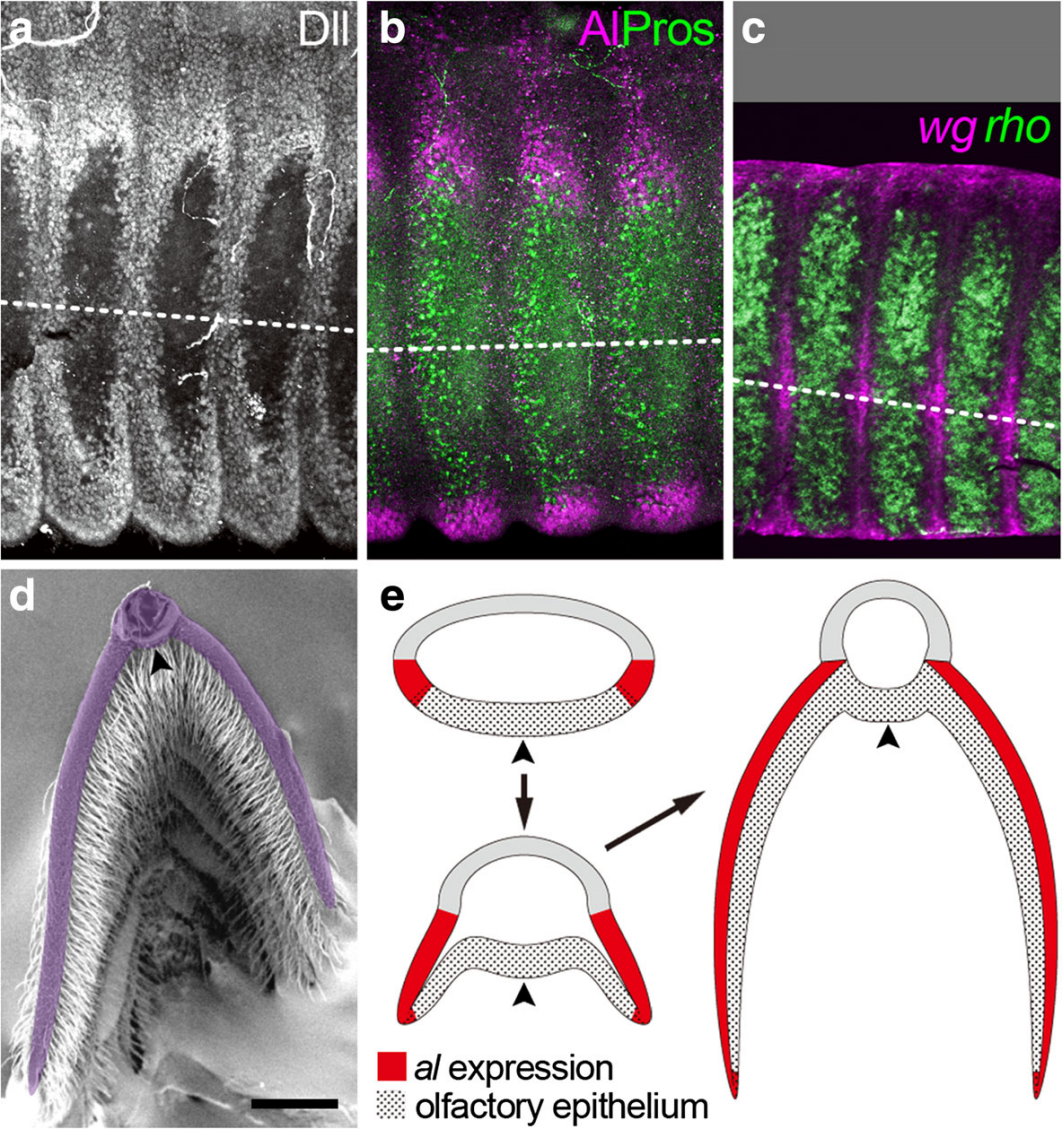
Expression patterns of antennal patterning genes in *B. mori*. (**a**) Dll expression in the early pupal antenna. Dll is not detected in the olfactory epithelium. **b** Al expression (magenta) in the early pupal antenna. The olfactory epithelium is labeled by a sensory organ marker, Prospero (Pros; green) [50]. Al expression is observed just dorsal to the olfactory epithelium and overlap with Dll expression. **c** *wg* (magenta) and *rho* (green) expression in the early pupal antenna. *rho* is expressed in the olfactory epithelium, while *wg* expression is detected between them. **d** SEM image of a *B. mori* antenna showing a cross-section (adapted from [35] with modification). **e** A schematic of cross-sections of a developing antenna showing the process of lateral branch formation. *al* expression is represented by red color and the olfactory epithelium by a dotted pattern. Broken lines in (**a**-**c**) and arrowheads in (**d**, **e**) indicate the ventral midline. Scale bar in (**d**) represents 130 μm in (**a**-**c**) and 200 μm in (**d**)

Olfaction is one of the most essential senses for animal survival. Accordingly, morphology and function of olfactory organs have been targets of natural selection in a wide range of animals. Terrestrial mammals with olfactory acuity, such as carnivores (e.g., cats and dogs) and ungulates (e.g., cattle and deer), have deeply convoluted olfactory epithelia [1, 2]. Nocturnal and cave-dwelling animals tend to have more sensitive olfactory organs than their diurnal or non-cave-dwelling relatives [3, 4]. In insects, the main olfactory organ is a pair of antennae on the head, which carry many odor-sensing organs on their surfaces. Antennae are segmented multiple times along the proximodistal (PD) axis and have a simple rod-shaped structure in many insect species. In Lepidoptera (moths and butterflies), however, those using pheromones for long-distance communication, such as nocturnal species, often have antennae with protrusions on or around the ventral side of each antennal segment. In extreme cases, such as *Bombyx mori* (silk moth), protrusions form remarkably long comb-like structures, which we term “lateral branches” (Figs. 1 and 2d). Since odor-sensing organs in such moths are densely packed and form the olfactory epithelium on the ventral side of each antennal segment, antennae with protrusions or elongated lateral branch structures can accommodate a wide surface area of the olfactory epithelium. In addition, protrusion structures aligned like a comb extend the time during which the pheromone or odorant plume passes through the antenna [5, 6]. These physical properties of protrusions or lateral branches in moths enhance the efficiency of olfactory reception and are believed to be ecologically important. In contrast, diurnal species of Lepidoptera, such as almost all butterflies, have simple antennae without protrusions or lateral branches (e.g. Fig. 1, *Papilio xuthus*; Additional file 1: Figure S1). Antennae with protrusions or lateral branch structures are thought to have originated from the filiform antenna of the primitive lepidopteran insects, such as micropterigid moths (e.g. Fig. 1, Micropterigidae gen.) and to have been acquired independently several times during the evolution of Lepidoptera (Fig. 1, Additional file 1: Figure S1) [7, 8].

Antennal development has been studied using branchless antennae of non-lepidopteran insects, including the fruit fly, *Drosophila melanogaster*. It has been suggested that antennal development along the PD axis is largely conserved: several transcription factor genes are expressed in specific regions along the PD axis and regulate the growth and differentiation of each region. Antennae are largely subdivided into proximal, medial, and distal parts by the combinatorial expression of *homothorax* (*hth*)/*extradenticle* (*exd*) and/or *Distal-less* (*Dll*) in each region [9–15]. The distal part, expressing only *Dll*, is further subdivided into several segments, and other transcription factor genes are expressed in the specific segment(s) among *Dll*-expressing segments. For example, *aristaless* (*al*) is specifically expressed in the most distal segment [15–17]. In *Drosophila*, according to studies of antennal development [9–11, 18] and the serial homology of antennae to legs [19–25], Wingless (Wg; a member of the WNT family), Decapentaplegic (Dpp; a member of the TGF-β family), and ligands of the epidermal growth factor receptor (EGFR) have been suggested to act as morphogens regulating the region-specific expression of these transcription factor genes along the PD axis in the antenna (for a review of leg development, see [26–29]). Wg and Dpp are expressed in the ventral and dorsal regions, respectively, as continuous stripes along the PD axis [30, 31]. EGFR ligands are thought to be produced from the distal tip as in the leg [32, 33]. Along with these PD patterning mechanisms, Notch signaling is also repeatedly activated along the PD axis and regulates antennal segmentation [34]. Although the understanding of antennal development along the PD axis has been advanced, the mechanism by which protrusions or lateral branches are formed in each antennal segment and how they have evolved remain largely unknown.

A previous study on the highly branched antenna of *B. mori*, which has two lateral branches per segment (Figs. 1 and 2d), has revealed patterns of gene expression, cell proliferation and cell death in lateral branch formation during metamorphosis [35]. Upon the onset of metamorphosis, many antennal patterning genes, including those involved in morphogen signaling, dramatically change their expression pattern from the conserved one described above to a segmentally reiterated, lateral branch-associated one. In the pupal antenna, each of these genes is expressed in a specific region within one segment, and the same expression pattern is repeated segmentally. Their expression patterns within each segment prefigure lateral branches. These expression patterns are not observed in other insects studied so far and a distinctive feature of the branch-associated antenna of *B. mori*. Importantly, just before the elongation of lateral branches, *Dll* expression disappears in the future olfactory epithelium, while it is strongly upregulated in the region surrounding the future olfactory epithelium and weakly in the remaining part (Fig. 2a) [35]. Furthermore, *al* is expressed in the two small regions just dorsal to both sides of the future olfactory epithelium (Fig. 2b) [35]. The *al* expression domains are included in the strong *Dll* expression domain (compare Fig. 2a and b). Each lateral branch then elongates during subsequent pupal development so that the region around the intersection between the *al* expression domain and the olfactory epithelium becomes the distal tip. Consequently, cells derived from the overlap between *Dll* and *al* expression contribute most of the dorsal side of each lateral branch, while the ventral side is covered by the olfactory epithelium (Fig. 2e) [35]. During the elongation of lateral branches, cells in the *al* expression domain abundantly proliferate, whereas cell death occurs almost ubiquitously. These observations predict that localized cell proliferation regulated by the branch-associated expression of antennal patterning genes is fundamental to lateral branch formation in the *B. mori* antenna. Furthermore, genes involved in morphogen signaling, such as *wingless* (*wg*) and *rhomboid* (*rho*; encoding an EGFR ligand activator), also show segmentally reiterated, branch-associated expression during metamorphosis (Fig. 2c) [35], implying their involvement in the regulation of antennal patterning gene expression within each antennal segment.

Here, we describe the results of functional analysis on *Dll* and *al* during lateral branch formation in the *B. mori* antenna. We also investigated the involvement of WNT, EGFR and Notch signaling in lateral branch formation. Our results show that the branch-associated expression of *al* and *Dll* is essential for lateral branch formation and that *al* expression is positively regulated by *Dll* and WNT signaling but restricted by Notch signaling. In addition, analyses of *al* expression in pupal antennae of several lepidopteran species, which are different in the extent of protrusions, revealed that the *al* expression pattern shows a striking correlation with antennal shapes, whereas the segmentally reiterated expression pattern was observed irrespective of antennal morphologies. Together with the results from functional analysis in *B. mori*, this observation implies that the variation in the *al* expression pattern within each antennal segment during metamorphosis is one of the significant determinants of antennal morphologies. According to these findings, we propose a mechanism underlying development and evolution of lepidopteran antennae with various morphologies.

## Results

### Requirement for *Dll* function in lateral branch formation

**Fig. 3:**
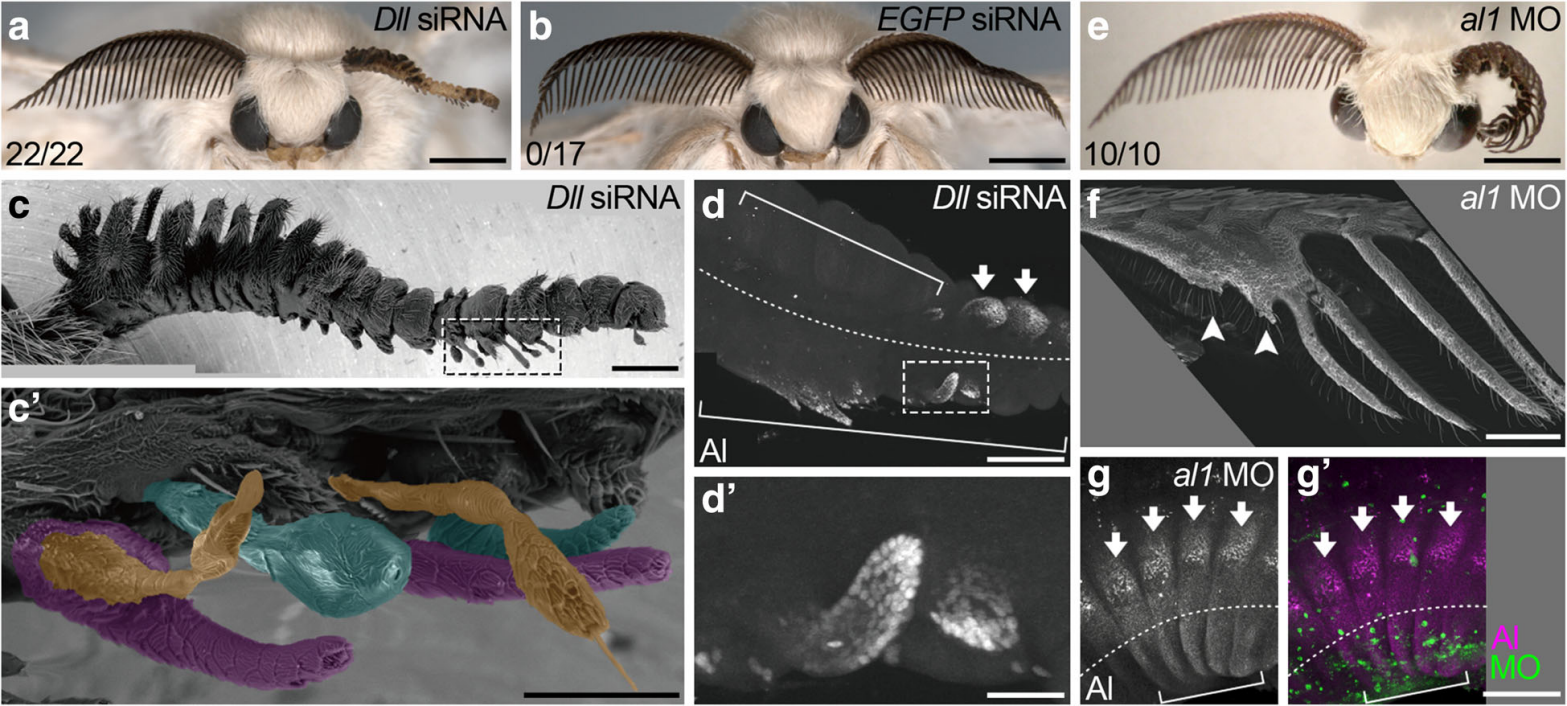
Requirement of *Dll* and *al* in lateral branch formation in the *B. mori* antenna. (**a**-**d’**) Adult antennae subjected to RNAi against *Dll* (**a**, **c**-**d’**) and *EGFP* (**b**). (**c**) A SEM image of a *Dll*-RNAi antenna showing lack or substantial shortening of lateral branches and relatively normal segmentation and segment length. (**c’**) The magnified view of another *Dll*-RNAi antenna showing the region similar to the region boxed by a broken line in (**c**). Note small and fine protrusions (pseudo colored). (**d**, **d’**) A pupal *Dll*-RNAi antenna stained by the anti-Al antibody. The region boxed by a broken line in (**d**) is magnified in (**d’**). *al* expression is lost in large area (bracket) and remaining *al* expression is associated with small protrusion. (**e**-**g’**) *al1*-MO antennae of adult (**e**, **f**) and pupa (**g**, **g’**). The anti-Al antibody signal is shown by white (**g**) or magenta (**g’**) colors. (**g’**) is an image merged with the fluorescein signal (green) of MO. The SEM image (**f**) shows loss or reduced lateral branches (arrowheads). The anti-Al antibody staining shows loss of *al* expression (bracket). Penetrations of defective antennae are shown at lower-left corner in (**a**, **b**, **e**). Dashed lines and arrows in (**d**, **g**, **g’**) indicate the ventral midline and normal Al expression, respectively. Scale bars represent 2 mm in (**a**, **b**, **e**), 500 μm in (**c**), 200 μm in (**c’**, **d**, **f**-**g’**), 50 μm in (**d’**)

**Fig. 4:**
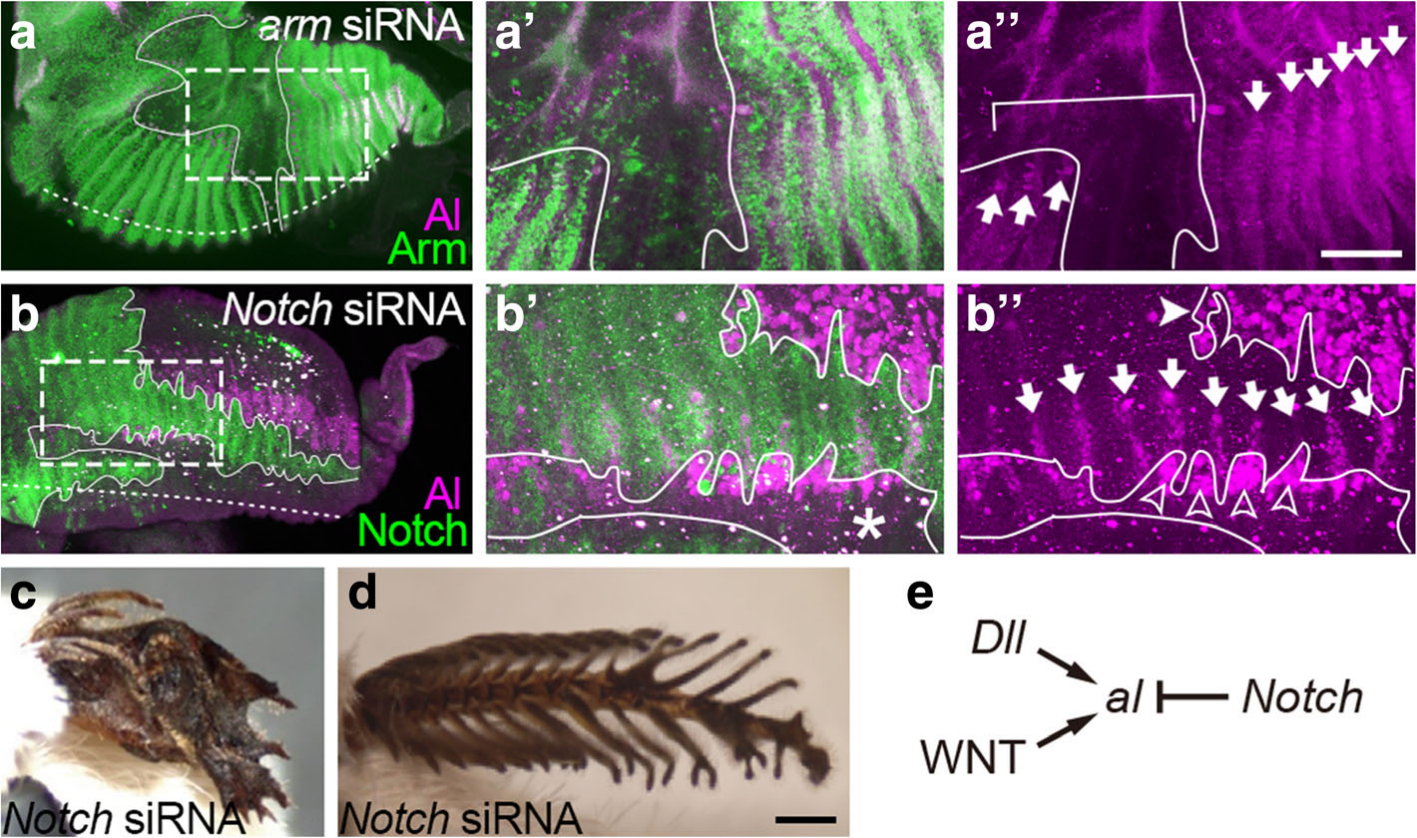
Regulation of *al* expression by WNT and Notch signalings in the *B. mori* antenna. (**a**-**a”**) An *arm*-depleted antennal primordium stained by the anti-Al (magenta) and Arm (green) antibodies. (**a’**, **a”**) Magnification of the region boxed by a broken line in (**a**). Only Al staining channel is shown in (**a”**). Al signals are not detected (bracket) in *arm*-depleted region (judged by the absence of the anti-Arm signals and surrounded by white line). (**b**-**b”**) An *Notch*-depleted antennal primordium stained by the anti-Al (magenta) and Notch (green) antibodies. (**b’**, **b”**) Magnification of the region boxed by a broken line in (**b**). Only Al staining channel is shown in (**b”**). In *Notch*-depleted region (judged by the absence of the anti-Notch signals and surrounded by white line), ectopic Al staining signals are detected in the region dorsal to (arrowheads) and between (open arrowheads) the normal Al expression domains. Note that the ectopic Al signals are not observed in the ventral region (asterisk). Arrows in (**a”**) and (**b”**) indicate the normal Al expression. (**c**, **d**) *Notch*-depleted adult antennae showing severe (**c**) and mild (**d**) phenotypes. Note that neighboring branches are fused in several regions in (**d**). (**e**) Possible regulatory interaction of *al* with *Dll*, WNT signaling and Notch signaling. Scale bar in (**a”**) represents 200 μm in (**a**, **b**) and 50 μm in (**a’**, **a”**, **b’**, **b”**). Scale bar in (**d**) represents 500 μm in (**c**, **d**)

To investigate *Dll* function specifically in lateral branch formation during *B. mori* antennal development, the electroporation-mediated RNA interference (RNAi) [36] was conducted. siRNAs against *Dll* (*Dll*-siRNAs) were injected and incorporated to developing antennal cells by the electroporation (see Methods). To specifically inhibit the segmentally reiterated, branch-associated expression during the pupal stage and minimize the defect in the basic PD development, we applied *Dll*-siRNAs 1 day before metamorphosis. All antennae subjected to *Dll*-RNAi (*Dll*-RNAi antennae), but not those subjected to RNAi against *Enhanced Green Fluorescent Protein* (*EGFP*) as a negative control, showed regions with missing or greatly reduced lateral branches (Fig. 3a, b). RNAi is expected to be induced only in cells incorporating enough siRNAs and thus, antennae subjected to RNAi possibly contain both RNAi induced and non-induced cells. It appeared, therefore, that lateral branches were missing or greatly reduced in the regions where *Dll* expression was depleted. In contrast to the substantial defects in lateral branches, segmentation appeared to be unaffected and segment sizes seemed relatively normal even in the region exhibiting extensive defects in lateral branch formation (Fig. 3C), although the overall length of antennae is somewhat reduced. This indicates that in our experimental condition, there is only a little, if any, influence on *Dll* function for the basic PD development and the defects in lateral branch formation were mainly resulted from the depletion of *Dll* expression in the pupal stage. Thus, *Dll* function during metamorphosis appears to be essential specifically for lateral branch formation. Interestingly, antibody staining using the anti-Al antibody revealed that the segmental expression of *al* was lost in many regions in *Dll*-RNAi antennae at the pupal stage (Fig. 3d). Therefore, *Dll* appears to positively regulate *al* expression within each segment during metamorphosis (see Fig. 4e).

### Significance of *al* function in lateral branch formation

Intriguingly, small and fine protrusions were frequently seen in the regions showing extensive defects in lateral branch formation in adult *Dll*-RNAi antennae (Fig. 3c, c’). Small protrusions, possibly corresponding to those observed in the adult antennae, were also found in pupal *Dll*-RNAi antennae (Fig. 3d, d’). The anti-Al antibody staining showed that they were always associated with the remaining *al* expression (Fig. 3d, d’). Together with the previous finding that the *al* expression domain within each antennal segment is the site of abundant cell proliferation and contributes to most of the dorsal side of the lateral branch [35], this tight association of *al* expression with the epithelial protrusion may suggest that the localized expression of *al* gives epithelial cells protrusive activity to elongate the lateral branch. If this is true, it is expected that the elimination of *al* activity would lead to the loss of the lateral branch. To test this, we knocked down *al* function specifically during metamorphosis with the electroporation of antisense morpholino oligomers against *al* (*al*-MO; see Methods). The anti-Al antibody staining signals were missing in several regions of pupal antennae subjected to *al*-MO (Fig. 3g, g’), confirming that *al* expression was removed in several regions by this treatment. In adult *al*-MO antennae, missing or greatly reduced lateral branches were observed (Fig. 3e, f, Additional file 2: Figure S2). These data indicate that the localized *al* expression within each antennal segment during metamorphosis regulates lateral branch formation, possibly by activating proliferation of *al*-expressing cells.

### Regulation of *al* expression within each antennal segment during metamorphosis by WNT signaling

The significance of the localized *al* expression within each antennal segment during metamorphosis for lateral branch formation led us to investigate the regulatory mechanism of *al* expression. Since *wg* and *rho* are also expressed in a segmentally reiterated pattern during metamorphosis [35], the involvement of WNT and EGFR signalings in the regulation of *al* expression within each antennal segment during metamorphosis were investigated by the electroporation-mediated RNAi. WNT signaling was attenuated by siRNAs against *armadillo* (*arm*), encoding a transducer of the canonical WNT signaling [37, 38], and EGFR signaling by siRNAs against *egfr*, a gene encoding EGFR itself [39]. Immunostaining of antennal primordia subjected to *arm*-RNAi with anti-Al and anti-Arm antibodies showed that *al* expression was lost in the *arm*-depleted regions (Fig. 4a-a”). In contrast, no significant change in *al* expression was observed in the regions where EGFR signaling seemed attenuated (judged by the lack of the anit-diphospholylated MAPK staining) in pupal antennae subjected to *egfr*-RNAi (Additional file 3: Figure S3). These results indicate that WNT signaling positively regulates segmental *al* expression during metamorphosis, whereas EGFR signaling is dispensable (Fig. 4e).

### Involvement of Notch signaling in determining *al* expression domain within each antennal segment

We next investigated the function of Notch signaling in the regulation of *al* expression during metamorphosis. Notch signaling has been known to regulate segmentation of antennae through segmentally repeated Notch activation in several insect species [15, 34, 40]. Accordingly, the involvement of Notch signaling in the regulation of segmentally reiterated *al* expression was expected. Notch signaling was attenuated by RNAi using the electroporation or lipofection to introduce siRNA against *Notch* (Additional file 4; Figure S4, see Methods). Adult antennae subjected to the electroporation-mediated *Notch*-RNAi showed a severe defect, in which most segments were drastically fused and the overall length along the PD axis was extensively reduced (Fig. 4c). This indicates that the involvement of Notch signaling in antennal segmentation appears to be conserved also in *B. mori*. In contrast to the severe case, the lipofection-mediated RNAi resulted in mildly affected antennae that showed branch fusion in several regions but without considerable reduction in the overall length (Fig. 4d). This suggests that neighboring lateral branches are segregated by Notch signaling. Antibody staining of such *Notch*-RNAi antennal primordia showed derepression of *al* in the *Notch*-depleted regions. *al* was ectopically expressed in the regions between its normal expression domains (Fig. 4b-b”, open arrowheads). These results are consistent with the above idea that *al* activates proliferation of *al*-expressing cells, and the fusion of neighboring branches in *Notch*-RNAi antennae can be explained as follows. Cells between branches ectopically express *al* and would abundantly proliferate in conjunction with flanking normal branch cells, resulting in the fusion of branches. In addition to the cells between branches, the ectopic *al* expression was also detected in the regions dorsal to its normal expression domains (Fig. 4b-b”, arrowheads). These results indicate that Notch signaling is required to repress *al* expression in the regions between and dorsal to its normal expression domains (Fig. 4e). Therefore, dorsal and lateral extent of the *al* expression domains within each antennal segment appears to be determined by this Notch-dependent repression. On the other hand, in the regions ventral to the normal *al* expression domains, which include both of the olfactory epithelia and regions between them, no ectopic *al* expression was observed (Fig. 4b-b”, asterisk), suggesting that the lack of *al* expression in these regions is independent of Notch signaling.

### Correlation between *al* expression pattern and antennal morphology in lepidopteran species other than *B. mori*

**Fig. 5:**
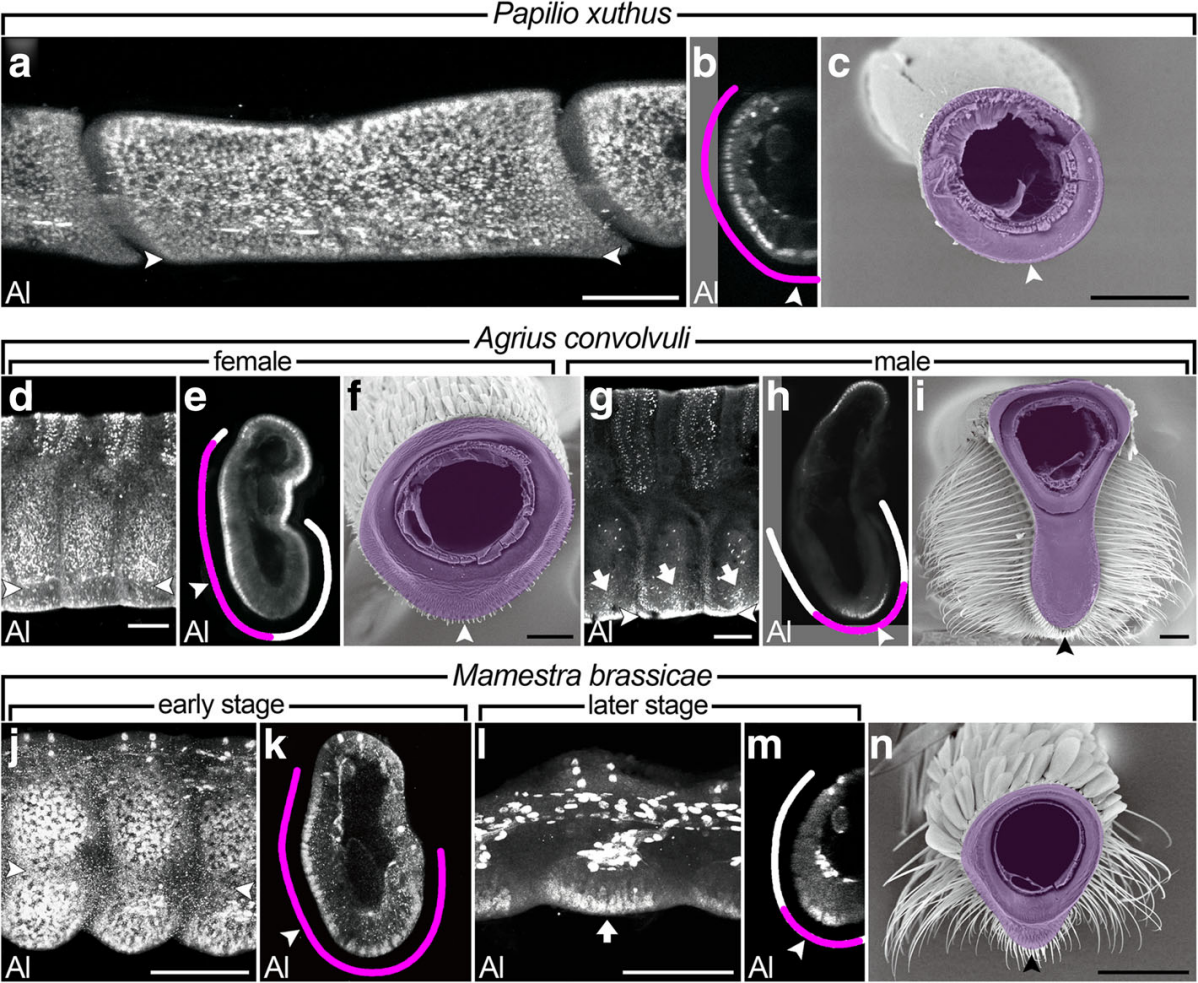
*al* expression patterns in pupal antennae of lepidopteran species with different antennal morphologies. Pupal (**a**, **b**, **d**, **e**, **g**, **h**, **j**-**m**) and adult (**c**, **f**, **i**, **n**) antennae of *P. xuthus* (**a**-**c**), female *A. convolvuli* (**d**-**f**), male *A. convolvuli* (**g**-**i**), and *M. brassicae* (**j**-**n**). (**a**, **d**, **g**, **j**) are surface views and (**b**, **c**, **e**, **f**, **h**, **i**, **k**, **m**, **n**) are cross-sections. (**l**) is a sagittal section. Pupal antennae are stained by the anti-Al antibody. Thick lines in (**b**, **e**, **h**, **k**, **m**) indicate the olfactory epithelium and Al-positive regions are represented by magenta color. Arrowheads in (**f**, **h**, **i**, **k**, **m**, **n**) and arrows in (**g**, **l**) indicate the ventral midline and the Al expression, respectively. Scale bars represent 100 μm

The significance of *al* function in lateral branch formation (see above) and localization of *al* expression to the region of abundant cell proliferation [35] in *B. mori* led us to examine *al* expression in pupal antennae of other lepidopteran species with various antennal morphologies in order to investigate the relationship between *al* expression patterns and antennal morphologies. We used three lepidopteran species, *Agrius convolvuli* (hawk moth), *Mamestra brassicae* (cabbage moth), and *Papilio xuthus* (swallowtail butterfly), all belonging to the same Lepidoptera subclade, Obtectomera, as *B. mori* (Fig. 1). The olfactory epithelium is formed on the ventral half of the antenna in all three species as in the *B. mori* antenna (Fig. 5c, f, i, n). *P. xuthus* has a simple antenna without branch structures or protrusions (Fig. 5c). In *A. convolvuli*, neither branch structures nor protrusions are observed in females (Fig. 5f). In males, the olfactory epithelium extensively protrudes ventrally, although no apparent branch structure is observed (Fig. 5i). *M. brassicae* has no branch structure but shows a slight protrusion around the ventral midline (Fig. 5n).

In all species examined, the anti-Al antibody staining of pupal antennae revealed that *al* is expressed in a segmentally reiterated manner as in the case of *B. mori* (Fig. 5a, d, g, j, l). Thus, segmental reiteration of *al* expression alone does not predict the antennal morphologies. Interestingly, however, there was a difference in *al* expression patterns within each antennal segment. In the *P. xuthus* antenna, which has no branch structure or protrusion, *al* was expressed broadly within each antennal segment (Fig. 5a, b). In the antenna of female *A. convolvuli*, which lacks branch structures or protrusions, *al* expression within each antennal segment was also detected broadly in the ventral region (Fig. 5d, e). In contrast, *al* expression in the pupal antenna of male *A. convolvuli*, whose adult antenna shows the extensive ventral protrusion of the olfactory epithelium, was restricted to the region around the ventral midline (Fig. 5g, h). In the *M. brassicae* antenna, showing modest ventral protrusion, *al* expression displayed a very interesting pattern. At the early pupal stage, *al* expression was observed in a broad region spanning almost the entire half of each segment (Fig. 5j, k). At the later stage, however, *al* expression was changed to be localized to the region around the ventral midline (Fig. 5l, m).

These observations clearly demonstrate that *al* expression patterns correlate well to antennal morphologies. Together with the functional analysis in *B. mori*, the striking correlation between *al* expression patterns within each antennal segment and antennal morphologies suggests the importance of the *al* expression pattern within each antennal segment in determining the extent of the association with branches or protrusions (see Discussion).

## Discussion

As pheromone-receptive organs, insect antennae have been diversified in their morphologies according to habitat environment, especially in Lepidoptera, from simple rod-like structures to multi-branched morphologies (Fig. 1, Additional file 1: Figure S1). Previous study on *B. mori* has revealed the unique pattern of gene expression and localized cell proliferation during lateral branch formation [35]. Yet, how lateral branches are formed in *B. mori* and various morphologies of antennae have been evolved are largely unknown. Functional analyses in *B. mori* described here strongly demonstrate that *al* expression within each antennal segment during metamorphosis is important for lateral branch formation. In addition, examination of *al* expression in other lepidopteran species with various antennal morphologies suggests possible importance of *al* expression pattern within each antennal segment on determining antennal morphologies.

### Regulatory mechanism to determine the *al* expression domain within a segment in the *B. mori* antenna

Our results indicate that lateral branch formation in the *B. mori* antenna is regulated by *al* possibly through its function in activating proliferation of *al*-expressing cells (Fig. 3e-g’). Therefore, determination of the *al* expression domain appears to be important for lateral branch formation. *Dll*-RNAi (Fig. 3d, d’) and *arm*-RNAi (4a–a”) experiments indicate that the segmental *al* expression during metamorphosis are positively regulated by *Dll* and WNT signaling (Fig. 4e). The *Dll* expression domain larger than the *al*-expressing region (Fig. 2a, b) [35] and the segmentally reiterated *wg* expression (Fig. 2c) [35] may suggest that *al* expression is activated in cells both expressing *Dll* and receiving an appropriate level of Wg. Since *arm* is a common downstream effector of several WNT family members, however, contribution of WNT other than Wg cannot be excluded. The *Notch*-RNAi experiment (Fig. 4b-b”) clearly shows that *al* expression is repressed by Notch signaling in the region dorsal to the *al* expression domains and the region between neighboring *al* expression domains (Fig. 4e). In the region ventral to the *al* expression domains, however, *al* expression was not derepressed even when Notch was depleted (Fig. 4b-b”). Given the requirement of *Dll* in antennal *al* expression within each antennal segment (Fig. 3d), the absence of *al* derepression from the olfactory epithelium may be due, at least in part, to the lack of *Dll* expression there (Fig. 2a) [35]. The absence of *al* derepression in the rest of the ventral region, in which *Dll* is expressed, can be explained if strong levels of Wg signaling repress *al* expression. The existence of other factors repressing *al* expression in the ventral region cannot be ruled out. Nonetheless, multiple regulatory inputs determine the strict expression domain of *al* within each segment in the *B. mori* antenna.

### Possible involvement of *al* in the formation of antennae with various morphologies

**Fig. 6:**
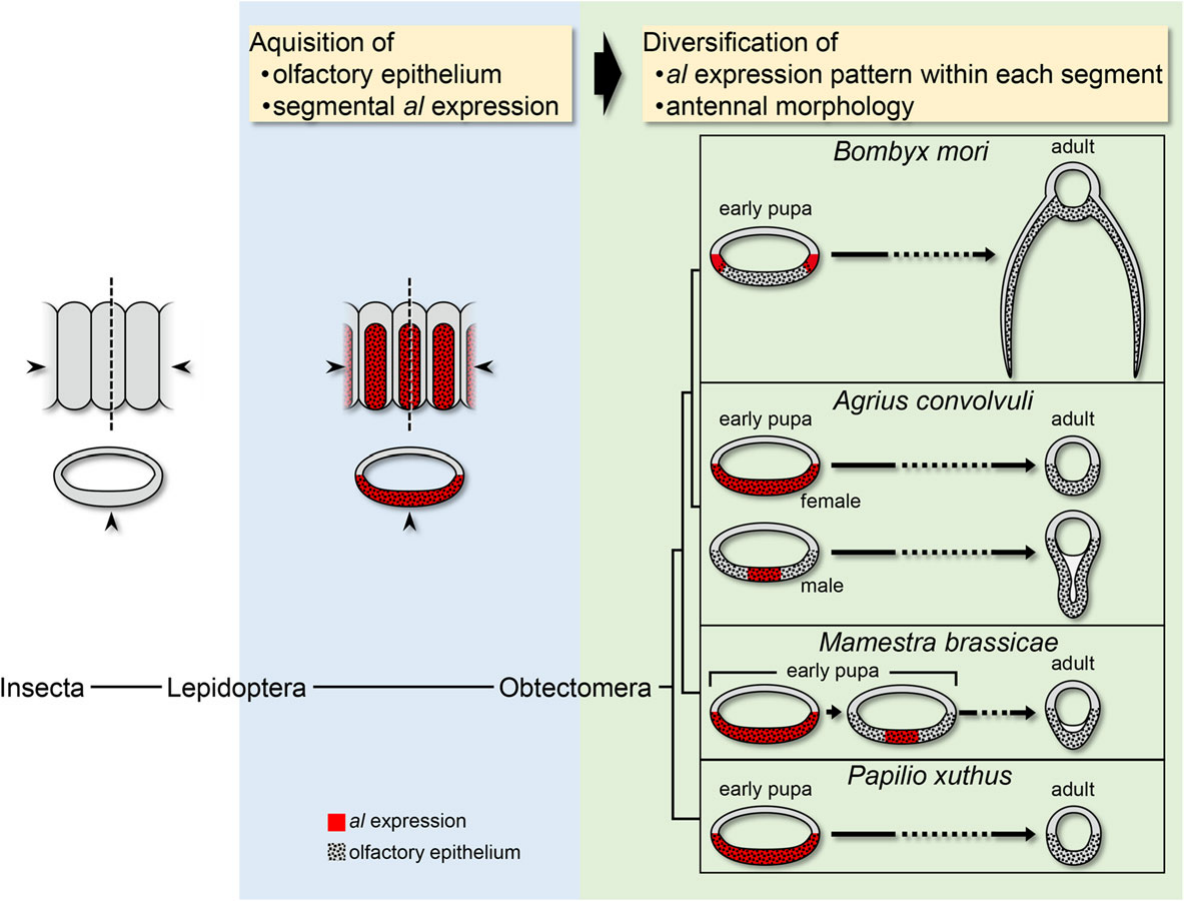
Possible mechanism underlying development and evolution of diverse antennal morphologies through modification of *al* expression. Ancestral insects may have had simple, rod-shaped antennae without protrusions or branches, and *al* may not have been expressed in a segmentally reiterated fashion. Protrusions or branch structures may have been acquired by the two-step change in *al* expression pattern. The first step is acquisition of segmentally reiterated *al* expression and its ventral restriction, as well as the olfactory epithelium with densely packed sensory organs, before the divergence of Obtectomera. This change in *al* expression, however, may not have changed antennal morphology. The second step is diversification of *al* expression pattern within each antennal segment, which may have led to the control of cell proliferation in specific regions and the development of protrusions or lateral branches after Obtectomera subclade had diverged. Arrowheads and dashed lines indicate the ventral midline and the position of cross-section, respectively. Red, the *al* expression domain; dotted region, the olfactory epithelium. See text for details

Assuming that *al* function suggested in the *B. mori* antenna is conserved among other species investigated in this study, the interspecific difference in antennal morphologies can be explained as follows. In male *A. convolvuli*, the restricted *al* expression around the ventral midline may induce cell proliferation and lead to the extensive ventral protrusion of the olfactory epithelium (Fig. 6, *Agrius convolvuli* male). In contrast, in *P. xuthus* and female *A. convolvuli*, cell proliferation would be possibly induced by the broad *al* expression in the region occupying almost the entire ventral half of antennal segments and this may lead to the uniform growth of the whole ventral olfactory epithelium so that the epithelial protrusion does not occur (Fig. 6, *Agrius convolvuli* female and *Papilio xuthus*). In *M. brassicae*, while cell proliferation possibly induced in the initial broad domain of *al* expression may not form an epithelial protrusion as in the cases of *P. xuthus* and female *A. convolvuli*, a later restriction of *al* expression to the region around the ventral midline may alter the pattern of cell proliferation, leading to slight protrusion of the ventral epithelium (Fig. 6, *Mamestra brassicae*). Another interpretation is also possible: the broad *al* expression in *P. xuthus* and female *A. convolvuli* may have little or no activity in regulating cell proliferation, whereas changes in *al* expression to the restricted region may be associated with the acquisition of function in controlling cell proliferation in *B. mori* and male *A. convolvuli*. In either case, a variation in the *al* expression pattern within a segment may strongly influence antennal morphology. Testing of these interpretations awaits further study in these species.

### Evolutionary perspectives of antennal morphologies

Considering that the branchless or non-protruding antennae of *P. xuthus* and female *A. convolvuli* seem to retain more ancestral characteristics of antennal morphology than the highly branched or extensively protruding antenna of *B. mori* or male *A. convolvuli* (Figs 2d and 5i), it seems reasonable to hypothesize that the broad expression of *al* is an ancestral state in the lineage leading to Obtectomera including these species (Fig. 6). Deviating from this ancestral situation, a subsequent restriction of *al* expression to two small regions within a segment may have led to the acquisition of two lateral branches on each segment in the lineage leading to *B. mori*, while its male-specific restriction to the region around the ventral midline in each segment may have resulted in the extensive ventral protrusion in males in the *A. convolvuli* lineage. Thus, changes in *al* expression within each antennal segment may have been important for morphological evolution of antennae.

Recently, it has been suggested that mutations relevant to the parallel evolution of morphology among different species are not randomly distributed in the genome, but occur in specific genes [41]. This conceptual gene, named an input-output gene, is expressed under the control of the integrated information from multiple upstream patterning genes and in turn regulates the expression of a battery of downstream effector genes involved in determining a final tissue structure [41]. Change in the expression pattern of the input-output gene by its regulatory mutations can alter the expression pattern of a battery of the downstream effector genes without any effects in the upstream patterning information. Thus, only the specific structure can be changed by regulatory mutations in the input–output gene without any effects on other structures. Such morphological change is expected to have minimal deleterious effects and if it is adaptive, associated regulatory mutations in the input–output gene can easily spread in the population [42]. As described here, *al* expression domains within each antennal segment during metamorphosis are variable with a pronounced correlation with antennal morphologies among different species in Lepidoptera. This correlation suggests that *al* is one of the input–output genes for antennal morphogenesis in Lepidoptera.

The segmentally reiterated expression of *al* observed in all of three species investigated in this study (Fig. 5a, d, g, j, l) suggests that *al*, and possibly other antennal patterning genes, had already been recruited to the segmentally reiterated expression pattern in an ancestor of the lineage leading to Obtectomera. Since the antennae of *P. xuthus* and female *A. convolvuli* lack branch structures or protrusions (Fig. 5c, f), however, this recruitment alone may not have led to the acquisition of branch structures or protrusions. It may have led to the uniform expansion of the olfactory epithelium and/or determination of the specific cell type(s) in a segmentally reiterated manner. Therefore, the acquisition of the segmentally reiterated expression appears to be cryptic from the viewpoint of antennal morphology but a prerequisite for further changes in the expression within each segment, which may have directly led to the formation of branch structures or protrusions. Once the segmentally reiterated *al* expression had been acquired, it might have been relatively easy to change its expression within a segment. This may be one of the forces facilitating the parallel evolution of antennal morphologies at least within, and possibly also outside of, Obtectomera. In light of the input-output gene concept, acquisition of the morphologically cryptic, segmentally reiterated expression in the ancestral lepidopteran species might have conferred on *al* the potential to be an input–output gene and thus, a target for evolutionarily relevant mutations, and allowed the parallel evolution of antennal morphologies in Lepidoptera. This stepwise mechanism may be one of general features of morphological evolution.

## Conclusions

In conclusion, our results show that in the *B. mori* antenna, *al* is essential for lateral branch formation and its expression domain appears to be determined strictly by the combinatorial function of *Dll*, WNT signaling and Notch signaling. Variation in the *al* expression patterns within each antennal segment, but not the segmentally reiterated expression itself, appears to be one of the significant factors for determining various antennal morphologies in Lepidoptera. Further research based on these findings will provide insights for understanding antennal evolution and general features of morphological evolution.

## Methods

### Insects

*B. mori* (N4 strain) and *M. brassicae* were reared on artificial diet (NIHON NOSANKO, Yokohama, Japan). *A. convolvuli* was reared on artificial diet containing sweat potato leaf extract. *P. xuthus* was reared on tangerine leaves. All insects were reared at 25 °C in long day condition (L:D = 16 h: 8 h) successively in our laboratory. *S. cynthia* was gifted from Dr. Z. Kajiura. Male animals were used for analysis unless otherwise noted.

### Developmental staging

The onset of pupal development (pupal molt) was monitored using a USB connection type CCD web camera equipped with an automatic infrared LED illumination system (GR-CAM130N2, Groovy, Tokyo, Japan). Pupal antennal primordia dissected at the stage when their epithelia were completely retracted from pupal cuticle were considered as samples at the early developmental stage (24 h after pupation [P24h] in *B. mori*, P72h in *M. brassicae*). Those dissected 12 or 48 h later before cuticle sclerotization were considered as samples at the late developmental stage (P84h in *M. brassicae*, P120h in *A. convolvuli* and *P. xuthus*).

### Antibody staining and in situ hybridization

Antibody staining and in situ hybridization was conducted essentially as described previously [35, 43]. The following antibodies were used: guinea pig anti-Al (1:1000) [44], goat anti-Dll (1:2, dF-20, Santa Cruz Biotechnology, Dallas, USA; pre-adsorbed with silkworm larval epidermal powder), rat anti-Pros (1:5, a gift from F. Matsuzaki), mouse anti-Notch (1:40, C17.9C6, Developmental Studies Hybridoma Bank, Iowa City, USA), mouse anti-Arm (1:40, N2 7A1, Developmental Studies Hybridoma Bank, Iowa City, USA), mouse anti-diphospho ERKI/II (1:250, M8159, Sigma-Aldrich, St. Louis, USA), and fluorophore (Alexa Fluor 488, 555, 647)-conjugated secondary antibodies (1:100, Thermo Fisher Scientific, Waltham, USA or Jackson ImmunoResearch, West Grove,USA). Riboprobes for in situ hybridization of *wg* and *rho* in *B. mori* were generated with DIG or Biotin RNA labeling kit (Roche, Basel, Switzerland) using cDNA as a template. Primers used are listed in Additional file 5: Table S1. The detailed procedures are described in Additional file 6.

### The electroporation- and lipofection-mediated RNAi

siRNAs were designed using siDirect version 2.0 [45] (Additional file 7: Table S2), and chemically synthesized and annealed (Fasmac, Atsugi, Japan). The electroporation-mediated RNAi was conducted as described previously [36]. In brief, 0.5 μL of 300 μM siRNA solution was injected through the tip of a larval antenna 1 day before metamorphosis. Immediately after injection, droplets of PBS were placed on the antenna and the lateral side of the head. Then, platinum electrodes were inserted into the PBS, and 5 square pulses of voltage (280 ms/s., 45 V) were applied. 24 h after the treatment, antennal primordia were collected for immunohistochemistry. We found that higher voltage or more pulses resulted in malformation of an antenna without siRNA.

In case of *Notch*-RNAi, the lipofection-mediated RNAi was conducted as a mild RNAi treatment. Lipofection mixture of siRNAs (61.25 μM each, Additional file 7: Table S2) was prepared by mixing TransFast transfection reagent (Promega, Madison, USA) and the same volume of siRNA solution. After incubating at room temperature for 15 min, 2 μL of lipofection solution was injected through the tip of an antenna of the late 4th instar (semifinal instar) larvae.

The adult antennae were photographed after eclosion under a stereomicroscope or a compound microscope.

We also tried to knock down *al* function by RNAi. There are at least two *al* homologs in the *B. mori* genome (*al1*/*BMgn006008* and *al2*/*BMgn006007*, SilkDB/KAIKObase). We designed four different siRNAs for each of *al1* and *al2*, and used them in various combinations. However, we failed to deplete either of *al1* or *al2* mRNA effectively by unknown reason (data not shown). Therefore, we tried using MO to knock down *al* function as described below.

### Morpholino oligomer (MO) treatment

The electroporation was used to incorporate MO as in the electroporation-mediated RNAi treatment, except that 0.5 μL of 1 mM MO solution was injected and 5 square pulses of voltage (280 ms/s., 41 V) were applied. After the treatment, the antennal primordia were collected for immunohistochemistry between P24h and P48h. The adult antennae were photographed after eclosion under a stereomicroscope.

To knock down *al* function, we designed MO against *al1*. Possible isoforms of *al1* mRNA in *B. mori* N4 strain was reconstructed by Trinity RNA-seq assembler on DDBJ Read Annotation Pipeline [46] using public RNA-seq data derived from female embryos 72 h after egg laying (DRR015667, NCBI Sequence Read Archive). We identified two isoforms with different translation initiation sites in the first exons (Additional file 8: Figure S5, Additional file 9: Figure S6). Their exon-intron structures were identified by mapping the mRNA sequences onto *B. mori* genome [47] using Exonerate software (ver. 2.2.0, with options ‘-m est2genome --score 1200’) [48]. Fluorescein labeled MO was designed at one of the two common splicing donor sites within the homeobox to skip the second exon encoding the polypeptide including N-terminal half of a homeodomain (Gene Tools, Philomath, USA; Additional file 8: Figure S5, Additional file 10: Table S3). Standard Control oligo with 3’ Fluorescein (Gene Tools, Philomath, USA; Additional file 10: Table S3) was used as a negative control. The electroporation with *al1*-MO resulted in loss of the anti-Al staining signals and inhibition of lateral branch formation (Fig. 3e-g’). Three possibilities can be considered to explain these observations: first, the anti-Al antibody could detect both Al1 and Al2 protein, and *al2* may not be expressed significantly in the antenna; second, the antibody used here may only detect Al1 protein but even if *al2* is expressed in the antenna, it may have no or little function in the lateral branch formation; third, there may be a cross regulation between *al1* and *al2* and knock down of *al1* alone may result in simultaneous downregulation of *al2*, leading to inhibition of both *al1* and *al2* activities. In any case, results of the MO experiment indicate that *al* activity for the branch formation is depleted successfully.

### Scanning electron microscopy (SEM)

SEM images were collected using scanning electron microscopes (Miniscope, Hitachi, Tokyo, Japan; JSM-5600LV, JEOL, Tokyo, Japan). Samples were attached to a pedestal with glue or nail polish without chemical fixation and analyzed according to the manufacturer’s instruction.

### Image processing

For the bright field images, images from several planes of focus were projected using Helicon focus (Helicon Soft, Kharkov, Ukraine) with Radius = 50 and Smoothing = 1. Brightness and contrast of the images were adjusted using Photoshop CS5.5 and CS6 (Adobe systems, San Jose, USA).

## Additional files

Additional file 1: Figure S1. Antennal morphology and phylogenetic tree of all lepidopteran families and superfamilies. The phylogenetic tree is based on the molecular phylogenetic estimation by Regier et al. [49]. Antennal morphology of each family was quoted from Scoble [8]. Antennal morphology of each family was categorized with the indicated color code in the upper left of the figure. The prominent bipectinate lateral branch appears to be acquired independently at least three times in the linage leading to swift moths (Hepialidae), bagworm moths (Psychidae), and the large group including Obtectomera and Cossoidea + Sessoidea + Zyaenoidea. Due to insufficient description or discrepancy between morphological classification and molecular phylogeny, several descriptions were redundantly quoted in distant families as below. (*) The same description in Papilionoidea was quoted. (**) The same description in Copromorphidae was quoted. (***) The same description in Megalopygidae was quoted. (****) The same description in Zygaenidae was quoted. (*****) The same description in Choreutidae was quoted. (TIFF 437 kb)

Additional file 2: Figure S2. Negative control of MO treatment. (A) Two classes of antennal morphologies (Normal, Defective) observed in the standard control morpholino treatment. Antennae with fused or short branches were categorized as “Defective”. Scale bar, 1 mm. (B) Distribution of Normal and Defective phenotypes in the standard control morpholino and *al1* MO treatments. The ratio of defective individuals in the *al1* MO treatment was significantly higher than that of the standard control morpholino treatment *, *p* = 3.1 * 10^− 3^ < 0.01, Fisher’s exact test. (TIFF 605 kb)

Additional file 3: Figure S3. Effect of reduced EGFR activity on *al* expression. Reduced EGFR activity was monitored using dpERKI/II signals. Segmentally reiterated EGFR activity was depleted in the region indicated with yellow arrowheads, whereas the native expression pattern of *al* (white arrowheads) was not affected, indicating that EGFR signal does not regulate induction of *al* expression at this stage. (TIFF 1275 kb)

Additional file 4: Figure S4. Statistical analysis of mild RNAi treatment against *Notch*. (A) The four categories of defects in lateral branch formation. Normal, lateral branches were not fused. I, fused lateral branches at one region. II, less than 3 subsegments were fused in several regions. III, 3 or more subsegments were largely fused. (B) Effect of *Notch* siRNA injection was compared with *Ubx* siRNA and Buffer injections. *Ubx* was selected as the negative control gene that is not expressed in the antenna. To conduct Fisher’s exact test, categories I to III were collectively categorized as “defective”. The ratio of defective individuals in *Notch* siRNA injection was significantly higher compared to the other two negative control experiments (*N* vs. *Ubx*, *p* = 3.6 * 10^− 8^ < 0.05; *N* vs. Buffer, *p* = 2.7 * 10^− 6^ < 0.05). (TIFF 686 kb)

Additional file 5: Table S1. Primers used for preparing RNA probes. (PDF 23 kb)

Additional file 6: Supplementary Methods. (PDF 350 kb)

Additional file 7: Table S2. Sequences of siRNA used in RNAi experiments. (PDF 271 kb)

Additional file 8: Figure S5. The exon-intron structure of *al1*. The two *al1* isoforms identified (*al1A*, *al1B*) have isoform-specific exons on the 5′ end (exon1), and share the remaining common exons (exon2-exon5). Homeobox is encoded in the region between exon2 and exon4. Orange indicates open reading frame. MO against *al1* was designed at the intronic region adjacent to the 3′ end of exon2 (MO target) to skip exon2. (TIFF 60 kb)

Additional file 9: Figure S6. The reconstructed exon sequences of *al1* mRNA isoforms. Yellow, open reading frame; pink, homeobox. (PDF 108 kb)

Additional file 10: Table S3. Sequences of Morpholino oligomers used to knock down *al1*. (PDF 29 kb)
