# Supplementary figures and images for: The pivotal role of aristaless in development and evolution of diverse antennal morphologies in moths and butterflies
Source: BMC Evol Biol. 2018 Jan 25;18:8. doi: 10.1186/s12862-018-1124-2 (PMC5785806; doi:10.1186/s12862-018-1124-2)

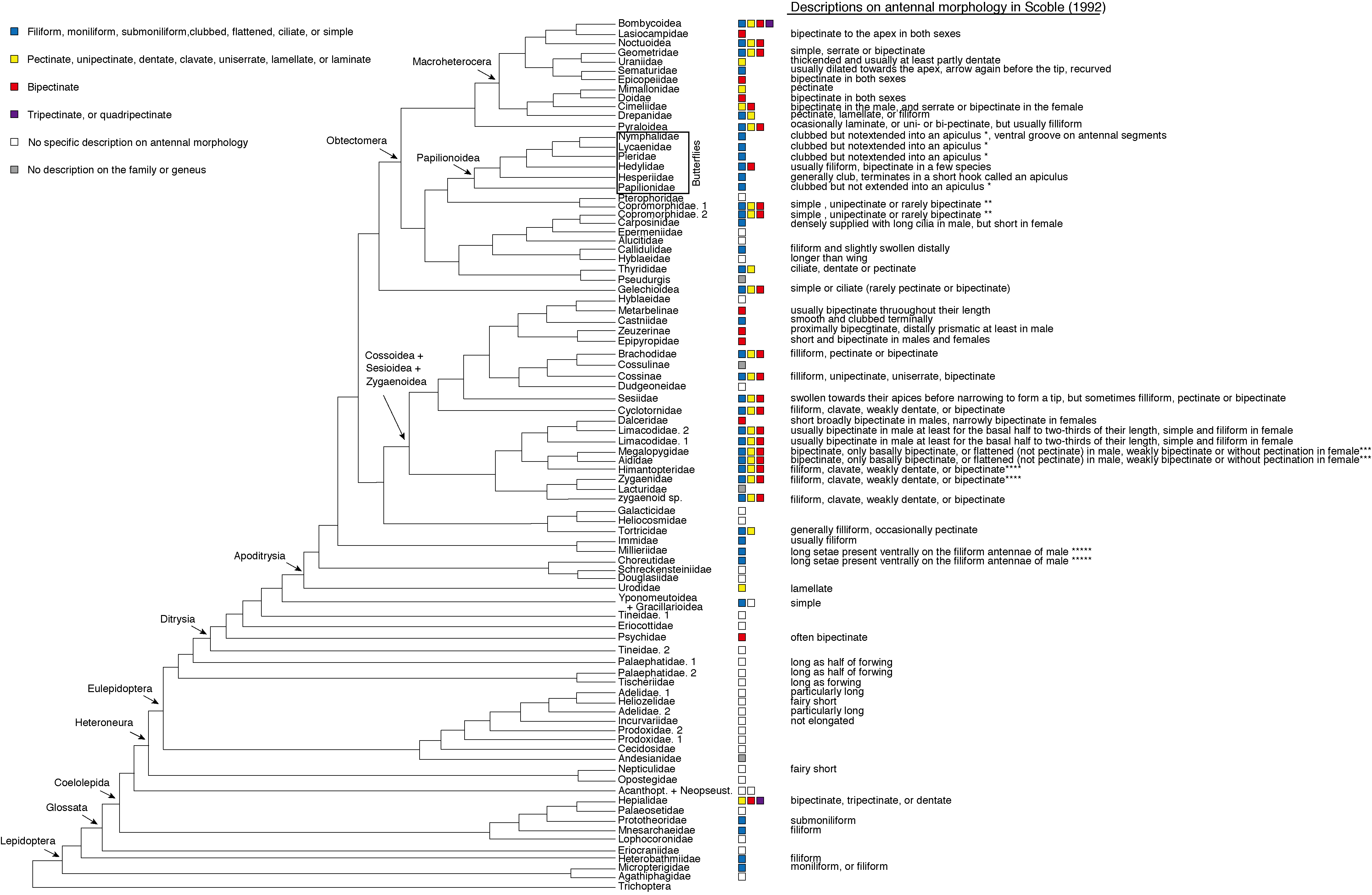

Supplement: Supplementary file 1 — Antennal morphology and phylogenetic tree of all lepidopteran families and superfamilies. The phylogenetic tree is based on the molecular phylogenetic estimation by Regier et al. [49]. Antennal morphology of each family was quoted from Scoble [8]. Antennal morphology of each family was categorized with the indicated color code in the upper left of the figure. The prominent bipectinate lateral branch appears to be acquired independently at least three times in the linage leading to swift moths (Hepialidae), bagworm moths (Psychidae), and the large group including Obtectomera and Cossoidea + Sessoidea + Zyaenoidea. Due to insufficient description or discrepancy between morphological classification and molecular phylogeny, several descriptions were redundantly quoted in distant families as below. (*) The same description in Papilionoidea was quoted. (**) The same description in Copromorphidae was quoted. (***) The same description in Megalopygidae was quoted. (****) The same description in Zygaenidae was quoted. (*****) The same description in Choreutidae was quoted. (TIFF 437 kb) [file 12862_2018_1124_MOESM1_ESM.tif]

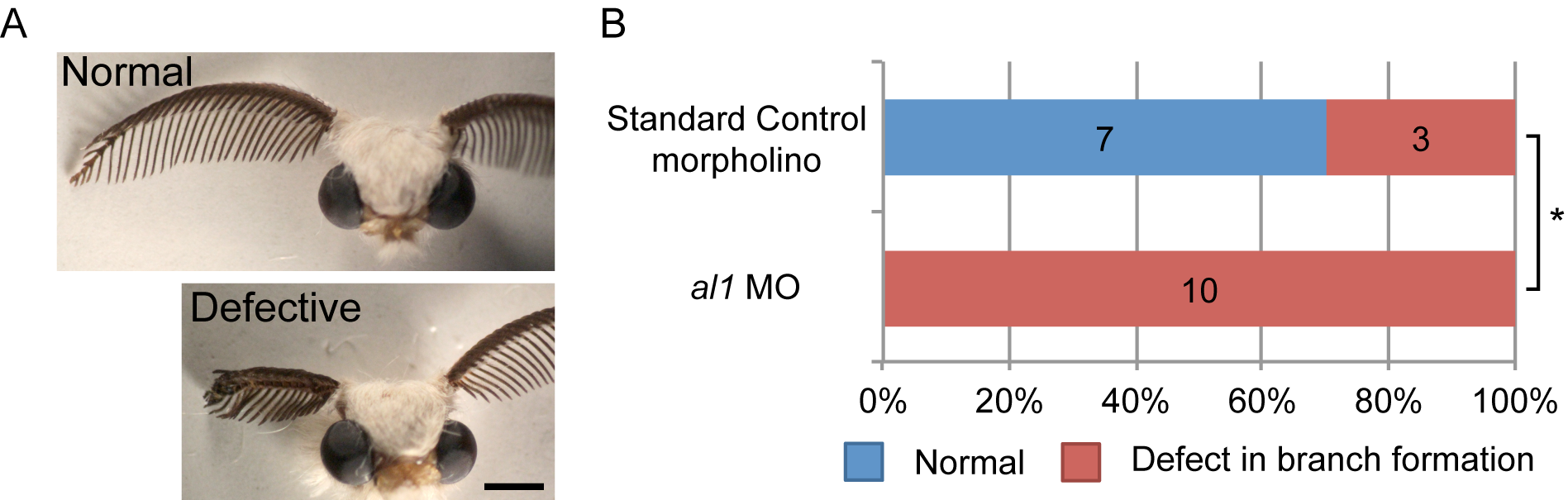

Supplement: Supplementary file 2 — Negative control of MO treatment. (A) Two classes of antennal morphologies (Normal, Defective) observed in the standard control morpholino treatment. Antennae with fused or short branches were categorized as “Defective”. Scale bar, 1 mm. (B) Distribution of Normal and Defective phenotypes in the standard control morpholino and al1 MO treatments. The ratio of defective individuals in the al1 MO treatment was significantly higher than that of the standard control morpholino treatment *, p = 3.1 * 10− 3 < 0.01, Fisher’s exact test. (TIFF 605 kb) [file 12862_2018_1124_MOESM2_ESM.tif]

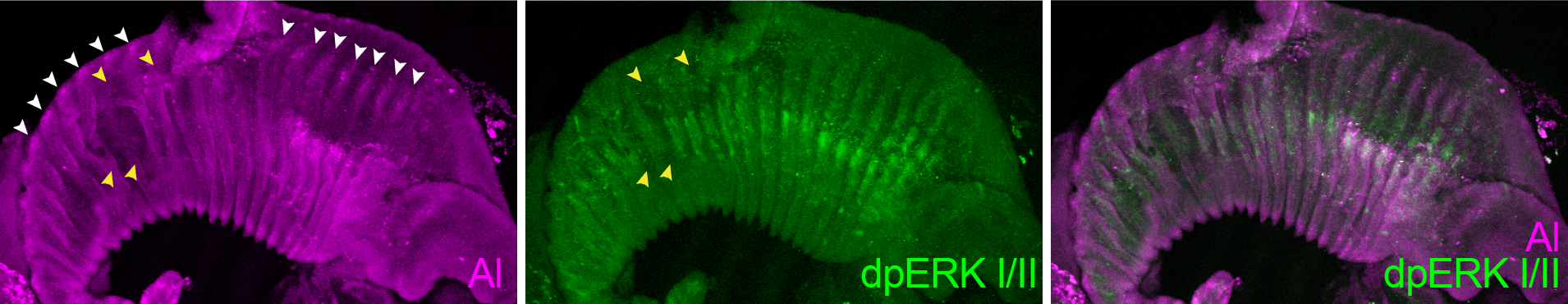

Supplement: Supplementary file 3 — Effect of reduced EGFR activity on al expression. Reduced EGFR activity was monitored using dpERKI/II signals. Segmentally reiterated EGFR activity was depleted in the region indicated with yellow arrowheads, whereas the native expression pattern of al (white arrowheads) was not affected, indicating that EGFR signal does not regulate induction of al expression at this stage. (TIFF 1275 kb) [file 12862_2018_1124_MOESM3_ESM.tif]

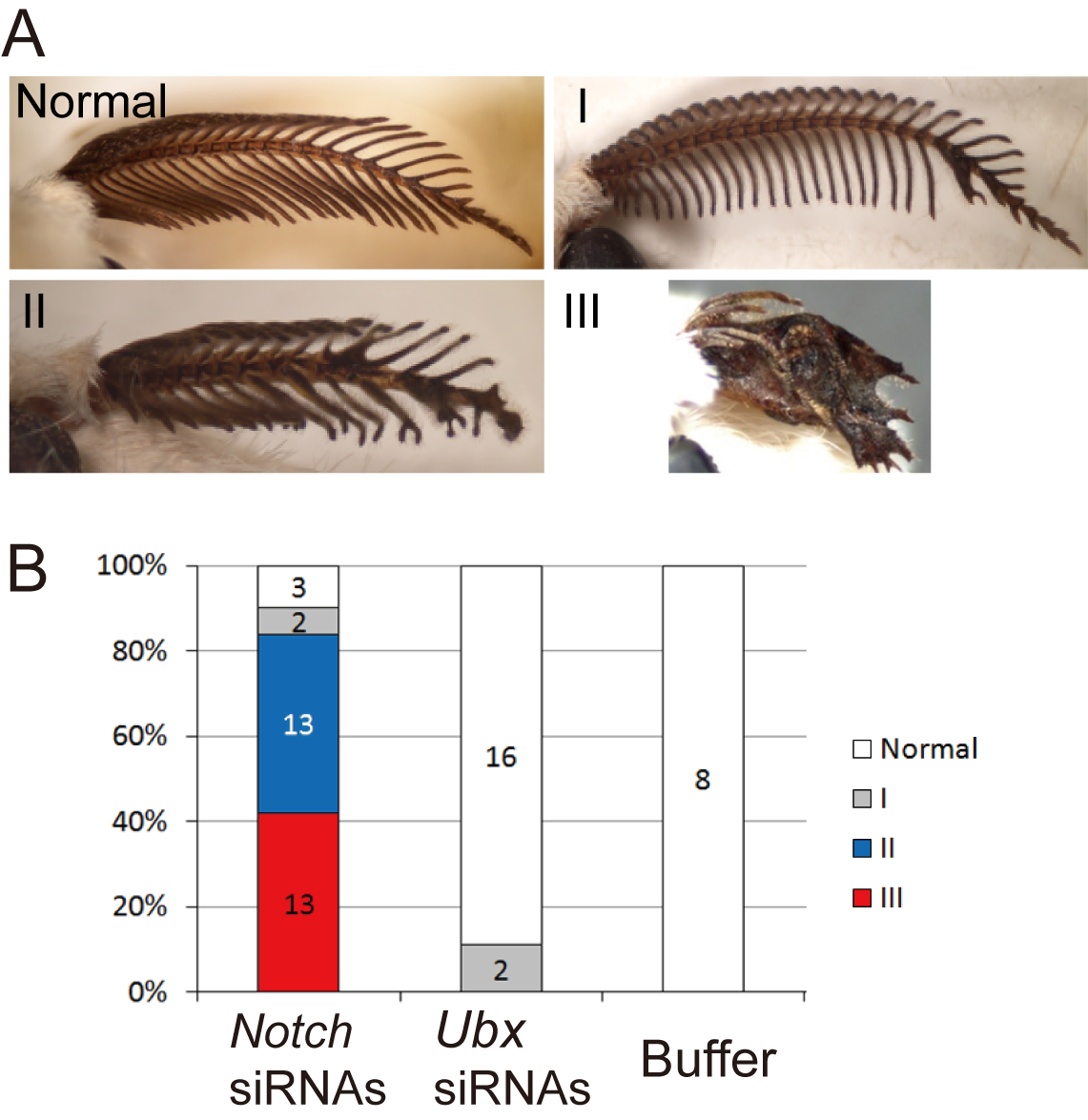

Supplement: Supplementary file 4 — Statistical analysis of mild RNAi treatment against Notch. (A) The four categories of defects in lateral branch formation. Normal, lateral branches were not fused. I, fused lateral branches at one region. II, less than 3 subsegments were fused in several regions. III, 3 or more subsegments were largely fused. (B) Effect of Notch siRNA injection was compared with Ubx siRNA and Buffer injections. Ubx was selected as the negative control gene that is not expressed in the antenna. To conduct Fisher’s exact test, categories I to III were collectively categorized as “defective”. The ratio of defective individuals in Notch siRNA injection was significantly higher compared to the other two negative control experiments (N vs. Ubx, p = 3.6 * 10− 8 < 0.05; N vs. Buffer, p = 2.7 * 10− 6 < 0.05). (TIFF 686 kb) [file 12862_2018_1124_MOESM4_ESM.tif]

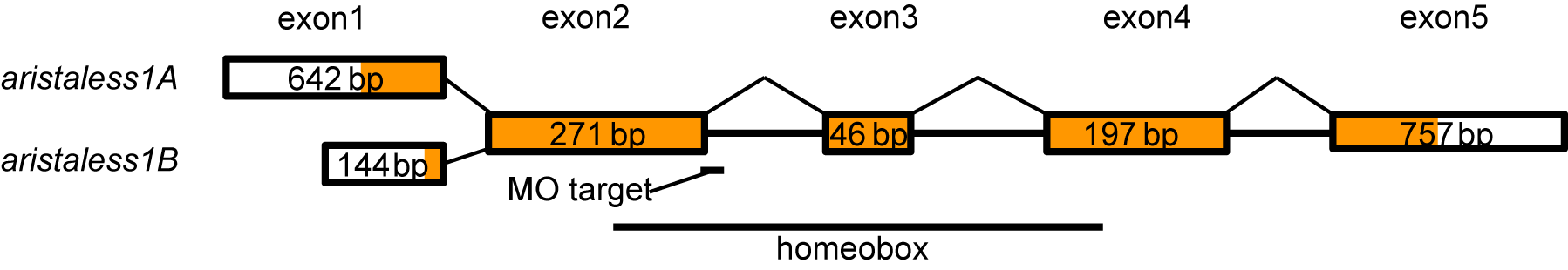

Supplement: Supplementary file 8 — The exon-intron structure of al1. The two al1 isoforms identified (al1A, al1B) have isoform-specific exons on the 5′ end (exon1), and share the remaining common exons (exon2-exon5). Homeobox is encoded in the region between exon2 and exon4. Orange indicates open reading frame. MO against al1 was designed at the intronic region adjacent to the 3′ end of exon2 (MO target) to skip exon2. (TIFF 60 kb) [file 12862_2018_1124_MOESM8_ESM.tif]
