# Supplementary material for: The pivotal role of aristaless in development and evolution of diverse antennal morphologies in moths and butterflies
Source: BMC Evol Biol. 2018 Jan 25;18:8. doi: 10.1186/s12862-018-1124-2 (PMC5785806; doi:10.1186/s12862-018-1124-2)
Supplement: Supplementary file 5 — Primers used for preparing RNA probes. (PDF 23 kb) [file 12862_2018_1124_MOESM5_ESM.pdf]

Table S1. PCR primers

Primer	primer sequence (5' > 3')	use
Bm_wg_S	ATGAAGTGTCTGTGGCTGTT	ORF cloning
Bm_wg_AS	TAATACGACTCACTATAGGGCTATAAACACGTGTGCACCACT	
Bm_rho_S	ATGAGTGGAAAGCGCACC	ORF cloning
Bm_rho_AS	TAATACGACTCACTATAGGGTCAGTAGCCGGCGTCG	
T7_Bm_wg_AS	TAATACGACTCACTATAGGGCTATAAACACGTGTGCACCACT	cRNA probe synthesis
T7_Bm_rho_AS	TAATACGACTCACTATAGGGTCAGTAGCCGGCGTCG	cRNA probe synthesis
