# Supplementary material for: The pivotal role of aristaless in development and evolution of diverse antennal morphologies in moths and butterflies
Source: BMC Evol Biol. 2018 Jan 25;18:8. doi: 10.1186/s12862-018-1124-2 (PMC5785806; doi:10.1186/s12862-018-1124-2)
Supplement: Supplementary file 6 — Supplementary Methods. (PDF 350 kb) [file 12862_2018_1124_MOESM6_ESM.pdf]

Supplementary Methods

Immunohistochemistry

Pupal and larval antennal primordia were dissected in phosphate buffered saline (PBS), and fixed with 4% paraformaldehyde (PA) in PBS for 30 min. Samples were permeabilized with PBS including 0.5% TritonX-100 for 30 min and washed with PBS including 0.01% Saponin (PBSS). After blocking with 1.5% blocking reagent (Roche, Basel, Switzerland) in PBS for 30 min, samples were incubated with primary antibodies for 1 hour. After 4 times of 10 min washes with PBSS, samples were incubated with fluorescence-labeled secondary antibodies for 1 hour. After 3 times of 10 min washes with PBSS, samples were mounted on slide glasses with VectaShield Mounting Medium (Vector Laboratories, Burlingame, California, USA), and analyzed with the scanning confocal microscope system (FV1000, Olympus, Tokyo, Japan).

Gene cloning

To obtain templates of RNA probe synthesis for *in situ* hybridization and dsRNA synthesis, gene fragments were cloned from cDNAs. Concerning *B. mori* genes, gene fragments (ORF of *wg* and *rho*) were cloned from cDNAs derived from polyA purified antennal mRNA at 5 days after the final larval molt. Briefly, the total RNA was extracted using TRI reagent (Sigma-Aldrich, St. Louis, Missouri, USA), and polyA RNA was purified using Micro-FastTrack 2.0 kit (Invitrogen, Carlsbad, California, USA). 150 ng of the polyA RNA was reverse transcribed with random primer (N_6) using First strand cDNA synthesis kit (GE healthcare, Chicago, Illinois, USA). Polymerase chain reaction (PCR) was performed using Ex-Taq (TaKaRa Bio, Otsu, Japan) and the gene specific primers designed based on the *Bombyx mori* genome sequence. The sequence information of the primers is listed in Additional file 5: Table S1. The amplified DNA fragments were cloned using pGEM T-Easy vector system (Promega, Madison, Wisconsin, USA). The DNA sequence was confirmed using BigDye terminator v3.1 Cycle Sequencing kit and genetic analyzer 3130*xl* (Applied Biosystems, Foster City, California, USA). All of the procedures were conducted according to the manufacturer’s instructions.

In situ hybridization

Template DNA fragments for RNA probe synthesis were amplified from the purified plasmids using the gene specific sense primers and gene specific, antisense primers fused with T7 promoter (*wg*, *rho*) (Additional file 5: Table S1). cRNA probes were synthesized using DIG (or Biotin) RNA labeling mix (Roche, Basel, Switzerland) and T7 RNA polymerase (TaKaRa Bio, Otsu, Japan). The cRNA probes used in fluorescent *in situ* hybridization were synthesized using cDNA fragments corresponding to

full-length ORF as templates, and were fragmented to approximately 500 bases under alkaline condition (41.9 mM $NaHCO_3$, 62.8 mM Na_2CO_3) at 60ºC for 10 min. To neutralize pH, 1/200 volume of glacial acetic acid was added and precipitated immediately using sodium acetate (pH 5.2) and ethanol. The concentration of RNA probes was measured using NanoDrop ND-1000 spectrophotometer (Thermo Fisher Scientific, Waltham, Massachusetts, USA). For fluorescent *in situ* hybridization, *Bm wg* probe was labeled with biotin, and *Bm rho* probe was labeled with DIG.

In situ hybridization was conducted as previously described [36]. Pupal antennal primordia were dissected in PBS, and fixed with 4% PA in PBS for 2 hours, and washed with PBS including 0.1 % tween20 (PTw). Fixed antennae were permeabilized with 0.5 % Triton X-100 for 45 min. After washing with PTw, samples were treated with 20 µg/mL proteinase K in PTw at 37ºC for 30 min. Proteinase K was immediately washed out by quick washes with 2 mg/ml glycine in PBS and following washes with PTw. We found that this proteinase K treatment is the most critical step for staining. After postfixation with 4% PA and 0.1 % glutaraldehyde for 30 min, samples were equilibrated to hybridization solution with 5 steps of hybridization solution series (50%, 75%, 87.5%, 100%, 100%) washes, and prehybridized at 57ºC, and hybridized with 100 ng/µL cRNA probes overnight (17h). After reverse hybridization solution series (50%, 25%, 12.5%, 0%[PTw] 0%[PTw]) and a wash with RNaseA reaction buffer (10 mM Tris-HCl, 500 mM NaCl, pH 8.0), single stranded probes not hybridized to mRNA were degraded with 20 µg/ ml RNaseA at 37ºC for 30 min. After equilibrated to hybridization solution again, nonspecifically bound degraded probes were washed out with 4 times of 20 minute washes with hot hybridization solution at 57ºC and following hot reverse hybridization solution series (50%, 25%, 12.5%, 0% [PTw]; 20 min for each). After washing with PBSS, immunohistochemistry was conducted using alkaline phosphatase-conjugated anti DIG Fab fragment (1:2000, Roche, Basel, Switzerland) for single color staining, or using streptavidin-HRP (1:2,500, Molecular Probes, Eugene, Oregon, USA) for double staining. Single color staining was conducted using BCIP/NBT solution kit (Nacalai tesque, Kyoto Japan). Coloration was conducted for 30 min to 6 hours according to the manufacturer's instruction. After washing with staining buffer and PBSS three times respectively, the solution was exchanged to 100% ethanol in two steps (50%, 100%), and the samples were de-colored for 1 hour. After returning to PBSS in 2 steps, the samples were mounted in 80% glycerol, and observed under complex microscope.

In double color staining, the first coloration was conducted using TSA kit #22 (Molecular probes, Eugene, Oregon, USA). According to the manufacturer's instruction, Tyramide labeling reaction was conducted for 40 min. The procedures below were conducted under a light shielding condition. After inactivating HRP activity with PBS including 1% H_2O_2 for 45 min, the samples were washed with

PBSS and stored at 4 ºC over night. Immunohistochemistry was conducted using alkaline phosphatase-conjugated anti DIG Fab fragment (1:1000) for 2 to 3 hours followed by 4 times of 15 min washes with PBSS. The second coloration was conducted using HNPP Fluorescent Detection Set (Roche, Basel, Switzerland). Coloration buffer and coloration solution was prepared according to the manufacturer's instruction. After washing with coloration buffer, samples were colored with the coloration solution for 1 to 3 hours. After washing with coloration buffer, fluorescence images were immediately acquired by the scanning confocal microscope system (FV1000, Olympus, Tokyo, Japan).
