# Supplementary material for: The pivotal role of aristaless in development and evolution of diverse antennal morphologies in moths and butterflies
Source: BMC Evol Biol. 2018 Jan 25;18:8. doi: 10.1186/s12862-018-1124-2 (PMC5785806; doi:10.1186/s12862-018-1124-2)
Supplement: Supplementary file 7 — Sequences of siRNA used in RNAi experiments. (PDF 271 kb) [file 12862_2018_1124_MOESM7_ESM.pdf]

Table S2. siRNAs

**siRNA**	**strand direction ***	**sequence (5' > 3')**	**Target position in ORF**
Dll	S	GAUCAUUAGGUUAUCCUUUCC	228-250
	AS	AAAGGAUAACCUAAUGAUCUA	
arm	S	GAACAAGUUGAUGAUAUGAUU	254-276
	AS	UCAUAUCAUCAACUUGUUCUU	
EGFR_A	S	UCACUUUUCCUUCUAAAGGUU	2012-2034
	AS	CCUUUAGAAGGAAAAGUGAAA	
EGFR_B	S	UUAAUUUGGCAAGAUUUGGUU	2666-2688
	AS	CCAAAUCUUGCCAAAUUAAGA	
*Notch*_A	S	AGUUAUUCCCGUAAAAGCCUU	4055-4077
	AS	GGCUUUUACGGGAAUAACUUU	
*Notch*_B	S	UGCAUUUUCUGUUGUCAAGUU	5039-5061
	AS	CUUGACAACAGAAAAUGCAUU	
*Ubx*_A	S	CCUAAAGCAGAUUGUUCAAUU	203-205
	AS	UUGAACAAUCUGCUUUAGGUU	
*Ubx*_B	S	GAGUUCCACACGAACCACUUU	530-552
	AS	AGUGGUUCGUGUGGAACUCUU	
