# Supplementary material for: The pivotal role of aristaless in development and evolution of diverse antennal morphologies in moths and butterflies
Source: BMC Evol Biol. 2018 Jan 25;18:8. doi: 10.1186/s12862-018-1124-2 (PMC5785806; doi:10.1186/s12862-018-1124-2)
Supplement: Supplementary file 9 — The reconstructed exon sequences of al1 mRNA isoforms. Yellow, open reading frame; pink, homeobox. (PDF 108 kb) [file 12862_2018_1124_MOESM9_ESM.pdf]

>Bm_aristaless_1A_exon1

CCTCGCAATCGCGGACTCAACGCGCGCTGAACGTTCACATCGCGAATAAAGCGCGAATA
GCCACACAAAATTCGGATACCGTTTGCCAAAAAAAAAACTCACAGTTGTTTTTGTTTCT
TTTTGACAGCTGTCAAATGAGTGAACGCTTTGTGACTGTATTGTGCAAGTGAGATCGAAA
GTGTCTGGCGCGACAAGAAAGCTCCGCGATACGACGATGACAATCGATTAAGCTATAATA
TTATTTTGATTTTTTTGTAAACAAAATATATAGTTCCATTATTTGAAATGCATTCTGATTGC
GATGCGCCCCGACTAATGTTCTTAGTTTAAAAATGTCAAAGGAAAACAGAAAGACATGTA
TGCGTTTATGTAGCGTTCTTCTGAACGTAAGTGCTTGTGAATATGGACTTGACGACTCGTC
GCGGGGATATGTTGGACGGAGCCGTCCCGGAATCTATATCAATGAACGGTTCGGATCAGT
GCCGAGATATTGTGAACGGACCCCAGAGCGGTAGTGACGTCACCGGTGTCACCGCCGAC
GTGCAGGTCCCTAGGAAGATTAACTCTTTCAGTATACGGAACTTGGTTGGCGGAGAGGA
CTCGAATCAGACAGCCGACGGAAACGTGACCGTAAATGATG

>Bm_aristaless_1B_exon1

GTTGTGAGTGCCGCAGCCTCGCACCCGCTCGCTTCTCCCCTTGTAATTAATTGATGTTTTC
ATTTAATAGCTTTCTCGGTAATTAGTTGTTAATATCTGAAATTGTGCACGTAAAGTTTTATC
GCGCTTGGTGATATTATATGTTGTTCCTGCAGCTACCGGACT

>Bm_aristaless_1_common_exon2

ATTATTATAACGAGCAGTCTTACCAGAAGTACTCAAGCATGGGCATATCGGAGGTGCCCA
AAGACGATTCCCCCCGGACCACTCCCGATCTATCTCGTACTGATCAGTCGCCTTCAGAAC
GACCACATCCAGGCTCCGTGGACAGCGATGATGCTGACGAATTTGCCCCGAAAAGGAAA
CAAAGGCGTTACAGAACCACCTTCACTAGTTTTCAACTTGAAGAGCTGGAGAAAGCGTT
TTCTAGAACCCATTATCCAGATGTATTTACGAG

>Bm_aristaless_1_common_exon3

GGAGGAGTTGGCAATGAAGATAGGATTGACTGAAGCTCGAATTCAG

>Bm_aristaless_1_common_exon4

GTCTGGTTCCAGAATCGACGGGCGAAGTGGAGGAAACAGGAAAAAGTTGGCCCCCAGG
GACACCCCTACAATCCTTACTTGAGCGCTACGGGAGCCGCCCCACCCGCCTCCGTGGTCG
CCTCCATGCCGAACCCCTTCACACAACTAGGGTTTGGTTTCAGGAAGCCCTTCGATACAA
ACGCTTTAGCTTCGTTTAG

>Bm_aristaless_1_common_exon5

GTATGCTGGGGGTCCAGTACTTGGAGCTCAGTATCTCGGTGCACCACTTCCACGACCACC
AATGTTCAGCGCCCCACTCTATACGAGTTCCCCCCCATTTCATTCCCTCCTCGCGGGTTTA
GCTCCTCCCCGACAATCTCCCGATCCGCCTCCTGTTTCTCCTCCTATATCTCCGGGCAGCG

AGTCTCCTCCTAACCAGCCTCCGGTTCAAGAAGTGGAGAGAAGAAGCTCGAGCATCGCA
GCCCTGAGGATGGCGGCTCGGGAGCACGAATTAAGATTAGAGATATTGAGGCAGCGACA
TCATAGCGACCTCATAAGTTGAGTTTGACTGTTATCCTAACATGGAAGACCAATTTTAACG
AATACCCAGGTCTTAATTAGCCAGATCTGCGATTTAATATAGGAGAAGACTGTTGGTTTTG
GTTTTGATTTTTAGTATTGAGAATTCTTACCGACAACGTTATGTTTCCTGTTATAATCAATA
GGTAATTATTAGAACACAAATTAGGCAGACACTACTTAAGTTTTCCGTGGTTTATCAGCGA
ATATGATGCAAATTCAGCCGCCTTAATAAGTTCCAGCTAAGTAGACTGACCAATCACTTGG
CATTCGTAATCTGTAATTGACTTAAGCACTTAACAACCAGTCAGCTTATTAATATACGTTAA
AACAGTTGAAACTTGTTTTACAGATTTGTATAGTAGAAAACATACTTCGTCTTCTCGCATT
AAAAGTGTATAATCTCAAAACTTACTAA
