# Supplementary material for: The pivotal role of aristaless in development and evolution of diverse antennal morphologies in moths and butterflies
Source: BMC Evol Biol. 2018 Jan 25;18:8. doi: 10.1186/s12862-018-1124-2 (PMC5785806; doi:10.1186/s12862-018-1124-2)
Supplement: Supplementary file 10 — Sequences of Morpholino oligomers used to knock down al1. (PDF 29 kb) [file 12862_2018_1124_MOESM10_ESM.pdf]

Table S3. Morpholino oligomers

Gene	Full name	sequence (5' > 3')	Length (bp)	Notes
al1	*aristaless1*	TTCTGCTATTTGTT ACCTACCTCGT	26	3' fluorescein
beta-globin (human, mutated form)		CCTCTTACCTCAG TTACAATTTATA	25	Standard Control oligo, 3' fluorescein,
